# Study the Association of Tumor Necrosis Factor Promoter Polymorphism with Type 2 Diabetic Nephropathy

**DOI:** 10.1155/2020/1498278

**Published:** 2020-07-01

**Authors:** Mahmoud Emara, Rawhia El-Edel, Waleed M. Fathy, Noran T. Aboelkhair, Mona M. Watany, Dalia H. Abou-Elela

**Affiliations:** ^1^Internal Medicine, Faculty of Medicine, Menoufia University, Egypt; ^2^Clinical Pathology, Faculty of Medicine, Menoufia University, Egypt; ^3^Clinical Pathology, Faculty of Medicine, Tanta University, Egypt

## Abstract

Type 2 Diabetes Mellitus (T2DM) is well known to include an inflammatory component that has been considered to be related to diabetic complications. Diabetic nephropathy (DN) is one of the significant complications as it constitutes the most frequent cause of end-stage renal disease. Tumor Necrosis Factor-*α* (TNF-*α*) is known as a multifunctional proinflammatory cytokine which is associated with some pathological processes such as immunoregulation, proliferation, inflammation, and apoptosis. The aim was to explore the association between the TNF-*α* promoter -*1031T*/*C* single nucleotide polymorphism (SNP) and the serum TNF-*α* level in addition to nephropathy among type 2 diabetic patients. The study included 38 T2DM subjects without nephropathy (DM group), 40 subjects with DN, and 20 controls. Identification of TNF-*α* promoter gene polymorphism -*1031T*/*C* was done by PCR-RFLP, and genotyping was confirmed by direct sequencing. The serum TNF-*α* level was assessed by ELISA. Correlations were tested by Pearson's correlation analysis. Logistic regression was used to detect the most independent factor for development of DN. The serum level of TNF-*α* in the DM group was significantly higher than controls (*p* < 0.001); also, the DN group was considerably higher than controls and DM without nephropathy (*p* < 0.001). Also, there was a significant positive correlation between serum levels of TNF-*α* with FBG (fasting blood glucose), creatinine, total cholesterol, LDL-C, HbA1c, and microalbumin/creatinine ratio (ACR) among the DN group (*p* = 0.042, <0.001, <0.001, <0.001, 0.027, and 0.043, respectively). Mutant homozygous CC and heterozygous TC genotypes were higher in DN than in DM and controls. C allele was more represented in DN than in DM and controls (*p* = 0.003) while T allele was higher in controls than in DM and DN patients. The levels of TNF-*α* were higher in subjects who had mutant CC than the wild TT genotype among DN (*p* < 0.001). C allele was more risky for DN than T allele between DN and controls by 5.4-fold (CI: 1.75-16.68) as well as between DN and DM by 2.25-fold (CI: 1.1-4.59). *Conclusion*. Serum levels of TNF-*α* were higher in individuals with mutant CC genotype of -*1031T*/*C* TNF-*α* gene, and C allele could be associated with increased risk for nephropathy among patients with T2DM.

## 1. Introduction

Type 2 DM is one of the most widespread metabolic disorders. Sustained hyperglycemia in patients with T2DM is the major cause of micro- and macrovascular complications including DN, which could develop at a later stage of the disease [1]. DN is considered the most common chronic microvascular complication of DM, and it seriously affects living quality of the patients. Inflammation and cell hypertrophy contribute to the progression of DN. The occurrence of DN is related to various factors including oxidative stress, high glucose, hemodynamic changes, and inflammatory processes [2]. DN was known as a nonimmune disease; however, evidences demonstrate overproduction of leukocyte adhesion molecules in kidneys in addition to an increase in macrophage infiltration [1].

Cytokines are significant mediators in the immune system, and their response due to an inappropriate balance may largely regulate disease predisposition. TNF-*α* is a cell signaling protein interrelated to systemic inflammation; in addition, it is a member of cytokines tangled in acute phase reaction [3]. TNF-*α* is produced mainly by monocytes/macrophages, even though intrinsic renal cells can also synthesize this cytokine. TNF-*α* actions are assisted by specific cell surface receptors. Binding of TNF-*α* to its receptors results in the expression of a variety of growth factors, transcription factors, cytokines, receptors, mediators of inflammatory processes, and acute phase proteins; in addition, it could mediate both apoptosis and necrosis. Consequently, TNF-*α* accelerates the release and synthesis of inflammatory mediators and could participate in the progress of DN [4, 5].

The locus of TNF-*α* gene is located within the Class III region of the human major histocompatibility complex (MHC) on chromosome 6 (6p21.31) traversing about 3 kb containing 4 exons. Control of TNF-*α* production takes place at both the transcriptional and posttranscriptional levels, with regulatory sequences within the 5′ end of the gene regulating the rate of transcription [6].

Many SNPs have been recognized in the promoter region of human TNF-*α* gene, having the ability to cause structural alterations inside regulatory sites that could disturb the function and regulation of TNF-*α* production. The location of the gene inside the MHC region has augmented the possibility that SNPs within this locus may contribute to the development of many diseases, such as T2DM and DN [7].

Polymorphisms located in the 5′ regulatory area of TNF-*α* gene at location -*1031T*/*C* have been associated with different inflammatory and autoimmune diseases [8, 9]. Therefore, this study is an attempt to explore the association between the -*1031T*/*C* SNP of TNF-*α* and the susceptibility to develop nephropathy in patients with T2DM.

## 2. Methodology

### 2.1. Subjects

This present case-control study involved 98 subjects who attended Internal Medicine Outpatient Clinics & Inpatient Department, Menoufia University Hospitals, Egypt, between September 2016 and December 2018. They were divided into 3 groups: group I: 20 apparently healthy controls; group II (DM): 38 T2DM without nephropathy, their albumin/creatinine ratio (ACR) < 30 mg/g; and group III (DN): 40 T2DM with nephropathy, their ACR < 30 mg/g.

Exclusion criteria were patients with history of hypoglycemic coma or diabetic ketoacidosis in the last 3 months before the study, urinary tract infection or other renal disease, hypertension, congestive heart failure, fever, inflammatory diseases like asthma and rheumatoid arthritis, pregnancy, infections, autoimmunity, neoplasm, or other endocrine diseases. This study was approved by the Research Ethics Committee, and written informed consent was obtained from all participants (written in Arabic).

### 2.2. Methods

For all subjects, the following were done: history taking, clinical examination, and assessment of anthropometric measurements.

#### 2.2.1. Biochemical Investigations

A fasting blood sample was withdrawn under aseptic protections and collected into plain and EDTA vacutainers. For glycated hemoglobin, 400 *μ*l of whole blood on EDTA was stored at 4–8°C, and estimation was carried out within 1 week of collection by ion-exchange resin chromatography using commercially available kits.

Another blood sample on EDTA was stored in aliquots at −80°C till further DNA extraction. Serum was separated for biochemical investigations such as fasting blood glucose (FBG), blood urea nitrogen (BUN), creatinine, and lipid profile, which were carried out using Beckman Coulter (Au 680) chemistry auto analyzer using a kit supplied by Beckman, USA, while low-density lipoprotein (LDL-C) was calculated by Friedewald's and Fredrickson's formula. An aliquot of serum was kept at −20°C till further use for estimation of the TNF-*α* level by ELISA. Another venous sample was taken after 2 hours of eating to measure the 2 h postprandial (2 h pp) glucose level. Urine samples were collected to estimate microalbumin and creatinine in urine, and then, ACR was calculated.

#### 2.2.2. DNA Extraction and Genotyping

Genomic DNA was extracted from whole blood using a commercially available DNA extraction kit (Thermo Fisher Scientific, USA). DNA was stored at −20°C until additional analysis. To genotype the -*1031T*/*C* polymorphism of TNF-*α* gene, polymerase chain reaction-restriction fragment length polymorphism (PCR-RFLP) was done.

**Figure 1:**
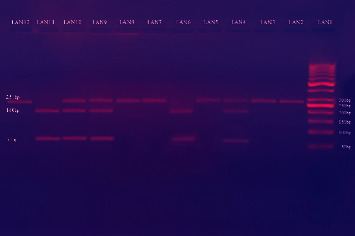
The products of TNF-*α* promoter gene after digestion by *BbsI* restriction enzyme on gel electrophoresis. LAN (1): 50 bp ladder; LAN (2, 3, 5, 7, 8, and 12): TT genotype; LAN (4, 6, 9, and 10): TC genotype; LAN (6 and 11): CC genotype.

These primers were used: forward 5′-TATGTGATGGACTCACCAGGT-3′ and reverse 5′-CCTCTACATGGCCCTGTCTT-3′. The total reaction volume was 25 *μ*l which included 5 *μ*l (100 ng) of genomic DNA, 4.5 *μ*l of nuclease-free water, 1.5 *μ*l forward and reverse primers, and 12.5 *μ*l of DreamTaq PCR Master Mix (Thermo Fisher Scientific , USA). Amplification was done using a Prime Thermal Cycler (Bibby Scientific Ltd., UK) with the following protocol: initial incubation at 95°C for 5 min, followed by 35 cycles of denaturation at 95°C for 30 s, annealing at 54°C for 30 sec, and extension at 72°C for 45 sec, followed by a final incubation at 72°C lasting 5 min. Amplicons with 264 bp in length were digested by *BpiI* fast digest restriction enzyme (Thermo Fisher Scientific, USA) incubated at 37°C for 15 min as a reaction mixture of 10 *μ*l of PCR product, 2 *μ*l of enzyme buffer, 1 *μ*l of the enzyme (BpiI) (1 U), and 7 *μ*l nuclease-free water. The digested products were separated by 2.5% agarose gel electrophoresis and identified by ethidium bromide staining. Stained fragments were visualized under UV (Figure 1); the fragments of 251 and 13 bp revealed homozygosity for the T allele and 180, 71, and 13 bp fragments indicated homozygosity for the C allele. Furthermore, genotyping results from the RFLP analysis were confirmed by direct DNA sequencing of the PCR products from each genotype in few samples, which were carried out on ABI PRISM™ 310 Genetic Analyzer using a kit supplied by Applied Biosystems, USA.

#### 2.2.3. Statistical Analysis

Data were collected, tabulated, and analyzed by SPSS software package version 20.0. For comparisons of demographic data and biochemical investigations between the studied groups, the following were used: chi-squared test, ANOVA test followed by post hoc Tukey's test, Kruskal-Wallis test followed by post hoc Dunn's test, Student's *t*-test, and Mann-Whitney test. The receiver operating characteristic curve (ROC curve) was performed to get the best cutoff for TNF-*α*. The Spearman coefficient was used for correlation. For genotype distribution, the chi-squared test and Monte Carlo test were used. Odds ratio (OR) and confidence interval (CI) were calculated by logistic regression analysis. The level of significance was set at 0.05 or less.

## 3. Results

**Figure 2:**
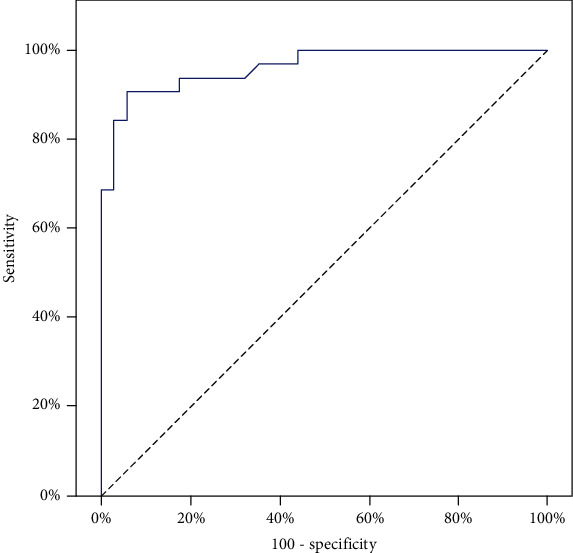
ROC curve for TNF-*α* to predict diabetic patients with albuminuria vs. diabetic patients without albuminuria.

The demographic data of the three groups are shown in Table 1. The body mass index (BMI) was considerably higher (*p* < 0.001) in DM and DN patients than controls. The duration of diabetes was longer in DN than DM. FBG, 2 h pp, and HbA1c were significantly higher (*p* < 0.001) in both patient groups compared to controls, where HbA1c was also higher in DN than DM (*p* = 0.013) (Table 1). Total cholesterol, triglyceride (TG), and LDL-C were significantly higher (*p* < 0.001) in DN patients as compared to DM, whereas the high-density lipoprotein (HDL-C) level is significantly lower (*p* < 0.001) in DN as compared to DM. Serum creatinine and BUN levels were significantly higher (*p* < 0.001) in DN compared to both DM and controls. As regards the serum TNF-*α* level, patient groups were significantly higher than controls; in addition, DN were significantly higher than DM (*p* < 0.001). The ROC curve (Figure 2) revealed that the best cutoff level of TNF-*α* was 155 ng/l, where sensitivity was 90.62%, specificity was 94.12%, positive predictive value was 93.5%, negative predictive value was 91.4%, and diagnostic accuracy was 92.42%. The TNF-*α* level showed a significant positive correlation with FBG, creatinine, HbA1c, total cholesterol, LDL-C, and ACR among the DN group (Table 2).

The genotypic and allelic frequencies of TNF-*α* gene promoter -*1031T*/*C* (Table 3) were found to follow Hardy-Weinberg equilibrium. Wild homozygous TT genotype was presented more in controls than DM and DN patients, whereas heterozygous TC and mutant homozygous CC genotypes were higher in DN than DM. Meanwhile, CC genotype was absent in controls, but this difference did not reach statistical significance. C allele was higher in DN than in DM and controls (*p* = 0.003), while T allele was more represented in controls than in DM and DN patients. The results also revealed that the TNF-*α* level in DM was significantly higher (*p* < 0.001) in TC and CC genotypes than TT genotype. Moreover, the TNF-*α* level among DN was significantly higher in CC genotype than TC and TT genotypes (*p* = 0.013 and <0.001, respectively). Also, TC genotype was significantly higher than TT genotype (*p* = 0.039). In addition, the C allele group among both DM and DN was significantly higher in the TNF-*α* level than the T allele group (*p* < 0.001).

The evaluation of the risk for DN at the genotype level is shown in Table 4. The TC genotype showed significant risk for nephropathy more than TT by 3.76-fold (CI: 1.03-13.69). At the same time, C allele was more risky than T allele between DN and controls by 5.4-fold (CI: 1.75-16.68), between DN and DM by 2.25-fold (CI: 1.1-4.59), and between patient groups and controls by 3.76-fold (CI: 1.27-11.18). Further, multivariate logistic regression for the risk factors of DN (Table 5) revealed that each C allele, duration of diabetes, and blood levels of BUN, creatinine, triglycerides, total cholesterol, LDL-C, HDL-C, HbA1c, and serum TNF-*α* carry the risk for DN; however, there was no independent risk factor as the disease is multifactorial.

## 4. Discussion

Diabetes is a promptly rising health problem in Egypt where the prevalence of T2DM was tripled in the previous 2 decades to reach around 15.6% of all adults 20–79 years [10]. Diabetic nephropathy as one of the most common diabetic complications affects up to 20–40% of patients with T2DM and may lead to end-stage renal disease, thus affecting the morbidity and mortality of these subjects [11]. Inflammation with high levels of proinflammatory cytokines such as IL-1, IL-6, and TNF-*α* is a significant feature of T2DM. These remarks suggest that TNF-*α* could participate in the pathogenicity of T2DM and DN. The TNF-*α* level varies among individuals; in addition, it is genetically determined [12].

Both patient groups were significantly higher in BMI than controls without significant difference between DM and DN. These results agreed with Doghish et al. [13] who reported that BMI did not differ between DM and DN patients. Meanwhile, Gupta et al. [1] reported that there was no significant difference observed in BMI between patient groups compared to controls.

Maric-Bilkan [14] stated that both obesity and diabetes share common initiating events which trigger intracellular signaling that initiates production of growth factors and cytokines, leading to renal illness.

The results revealed that the duration of diabetes was longer in DN than DM. This agreed with Mahfouz et al. [15]; meanwhile, Ochodnicky et al. [16] and Motawi et al. [17] reported that there was no significant difference in the duration of diabetes between DM and DN. The relation between DN and duration of diabetes was explained by Gallagher and Suckling [18] who reported that prolonged exposure to hyperglycemia causes damage to kidney structures, either directly or through hemodynamic changes. Also, Anders et al. [19] stated that hyperglycemia lowers sodium exposure at the macula densa, which inhibits tubuloglomerular feedback, dilates the afferent arteriole, and induces glomerular hyperfiltration, triggering podocyte barotrauma and resulting in podocyte and nephron loss.

In the present study, FBG, 2 h pp, and HbA1c were significantly higher in patients compared to controls, while there was no significant difference between the DM and DN groups. This agreed with Motawi et al. [17]; meanwhile, Alnaggar et al. [20] and Gupta et al. [1] found that FBS and 2 h pp were significantly higher in DN compared with DM. Saulnier-Blache et al. [2] reported that HbA1c did not differ between DM and DN. Hyperglycemia has been considered the initiator of renal pathology associated with DN by deregulation of numerous metabolic pathways. It was reported that hyperglycemia leads to an increase in oxidative stress by exacerbating mitochondrial generation of reactive oxygen species which cause DNA damage contributing to apoptotic cell death [21].

This study had found that lipid profile obtained results agreed with Mahfouz et al. [15]. Meanwhile, Alnaggar et al. [20] reported that there was no major difference between DM and DN regarding lipid profile, as dyslipidemia increased extracellular matrix expression and macrophage activation in the glomeruli in diabetic conditions, leading to DN. Doghish et al. [13] reported that dyslipidemia has been found in T2DM patients with early kidney injury. It is due to impaired function of lipoprotein lipase which is located in the endothelial cells, leading to raised TG and decreased HDL-C.

Our results revealed that the serum levels of TNF-*α* were significantly elevated in both patient groups than controls; in addition, DN were higher than DM. The TNF-*α* level showed a significant positive correlation with FBG, creatinine, total cholesterol, LDL-C, HbA1c, and ACR in the DN group. These results are consistent with those observed by Navarro and Mora-Fernandez [4] and Chen et al. [3] who also found elevated TNF-*α* levels in DN patients suggesting an elevated inflammatory milieu in DN. In addition, patients with T2DM had been reported to have 3–4 times greater TNF-*α* circulatory levels compared to controls, and these levels are more elevated in diabetic patients with microalbuminuria with respect to normoalbuminuria. Centered on the above, the results support the cytotoxic role of TNF-*α* in the glomerular damage mediated by hyperglycemia, which in turn leads to progressive albuminuria [22].

Moreover, Umapathy et al. [6] found that the serum TNF-*α* level showed a positive correlation with age and urea and a negative correlation with HDL-C and LDL-C. However, Gupta et al. [1] reported that the TNF-*α* level in both patients and controls did not reach statistical significance. This could be due to the discrepancies in their study designs and variations in the genetic background and may also be due to environmental exposures of the T2DM patients enrolled in these studies.

The TNF-*α*—mainly produced by monocytes and macrophages—plays a vital function in the course of DN. Also, it had effects on insulin resistance and insulin secretion. In addition, TNF-*α* has been cytotoxic to the epithelial, mesangial, and glomerular cells that could lead to direct kidney injury. Also, it was stated that TNF-*α* had a critical role in mediating inflammatory processes, which was involved in glomerular and tubulointerstitial damage [23].

SNPs in the TNF-*α* gene have a direct functional importance in terms of regulating TNF-*α* production. Particularly, there is a concern in these polymorphic sites in the regulatory regions of the TNF-*α* gene that relate with the DNA motifs in which the transcription factors bind. The -*1031T*/*C* polymorphisms in the 5′ regulatory region of TNF gene have been associated with many inflammatory and autoimmune diseases. To clarify the TNF gene polymorphism role in the pathogenesis of DN, the -*1031T*/*C* SNP was studied in DM and DN then interrelated to TNF-*α* level.

The results revealed that homozygous mutant CC genotype had an elevated level of serum TNF-*α* among DN patients than the subjects with wild TT genotype; in addition, the serum TNF-*α* level was found to be higher in both DM and DN individuals of C allele compared to T allele.

A previous study conducted by Gupta et al. [1] revealed that C allele of TNF-*α* gene promoter -*1031T*/*C* polymorphism was associated with elevated plasma TNF-*α* levels; however, this was not statistically significant. They also found that T2DM patients having T/C and C/C SNPs had 3-fold increased risk of evolving nephropathy. This discrepancy in the results could be the variation in the ethnicity of the study groups.

It has been well documented that the homozygous CC genotype of TNF-*α* is a vital marker in few inflammatory diseases being a high producer of TNF-*α* and disease susceptibility. The C variant stimulates the binding of nuclear factors except NF-*κ*B to the TNF-*α* gene promoter, which may lead to a rise in TNF-*α* expression [24]. This increased level is due to the response of stimulating factors such as inflammation, in which both TNF-*α* and its receptors are expressed and stimulate the production of other cytokines such as IL-8, acute phase proteins, chemokine, and growth factors by adjacent cells. Further, TNF-*α* has been reported to participate in the development of DN through several mechanisms, including the decreased glomerular filtration rate and the glomerular blood flow and disturbance of the glomerular filtration barrier. Increased production of TNF-*α* can stimulate oxidative stress and have a direct cytotoxic and apoptotic effect on glomerular cells [6].

In summary, this study exposed that the C allele of -*1031T*/*C* SNP of TNF-*α* is concomitant with a significant risk for development of diabetic nephropathy. Also, the circulatory levels of TNF-*α* were higher in individuals with mutant genotype. The strength of the present study is that all patients and controls are of the same ethnic origin. We conclude -*1031T*/*C* SNP of TNF-*α* to be a genetic vulnerability factor for DN, which would attribute to the anticipation and early diagnosis of DN. However, further studies among different ethnic populations and with large sample size are necessary to attain more evidence.

## Figures and Tables

**Table 1:** Demographic data and biochemical tests.

Parameters	Control group (*n* = 20)		T2DM group (*n* = 38)		DN group (*n* = 40)		Test of sig.	*p*	Post hoc test		
									Controls vs. T2DM	Controls vs. DN	T2DM vs. DN
	No.	%	No.	%	No.	%					
Gender							*χ* ^2^ = 0.021	0.989	—	—	—
Male	15	75.0	28	73.7	30	75.0					
Female	5	25.0	10	26.3	10	25.0					
Age (years)							*F* = 0.022	0.978	—	—	—
Min.–Max.	36–65		36–63		36–65						
*X* ± SD	47.30 ± 8.66		47.76 ± 8.23		47.50 ± 8.41						
BMI (kg/m^2^)							*H* = 45.164^∗^	<0.001^∗^	<0.001^∗^	<0.001^∗^	0.132
Min.–Max.	24.3–29.1		27.5–33.7		29.1–33.1						
Median	28.10		30.45		31.1						
Duration diabetes (years)	—						*t* = 10.415^∗^	<0.001^∗^	—	—	—
Min.–Max.			3.0-9.0		7.0-20.0						
Mean ± SD			6.37 ± 1.46		12.35 ± 3.31						
FBS (mg/dl)							*H* = 47.388^∗^	<0.001^∗^	<0.001^∗^	<0.001^∗^	0.747
Min.–Max.	65.0–99.0		107.0–370.0		107.0–360.0						
Median	81.0		155.0		164.50						
2 h pp (mg/dl)							*F* = 91.362^∗^	<0.001^∗^	<0.001^∗^	<0.001^∗^	0.269
Min.–Max.	108.0–139.0		210.0–401.0		190.0–455.0						
*X* ± SD	122.4 ± 9.24		274.58 ± 48.46		291.45 ± 57.39						
BUN (mg/dl)							*F* = 100.298^∗^	<0.001^∗^	0.490	<0.001^∗^	<0.001^∗^
Min.–Max.	6.0–21.0		6.0–31.0		19.0–96.0						
*X* ± SD	13.45 ± 4.51		17.58 ± 6.31		54.08 ± 19.19						
Creatinine (mg/dl)							*H* = 63.675^∗^	<0.001^∗^	0.319	<0.001^∗^	<0.001^∗^
Min.–Max.	0.40–1.30		0.50–2.0		1.30–7.10						
Median	0.80		0.90		1.85						
TG (mg/dl)							*F* = 34.876^∗^	<0.001^∗^	0.282	<0.001^∗^	<0.001^∗^
Min.–Max.	72.0–192.0		36.0–195.0		109.0–275.0						
*X* ± SD	114.25 ± 31.48		129.92 ± 40.16		187.15 ± 36.64						
Total cholesterol (mg/dl)							*H* = 50.384^∗^	<0.001^∗^	0.093	<0.001^∗^	<0.001^∗^
Min.–Max.	119.0–198.0		103.0–426.0		168.0–411.0						
Median	166.50		182.50		266.0						
HDL-C (mg/dl)							*F* = 51.967^∗^	<0.001^∗^	<0.001^∗^	<0.001^∗^	<0.001^∗^
Min.–Max.	40.0–56.0		10.0–51.0		7.0–49.0						
*X* ± SD	46.50 ± 4.73		34.79 ± 8.62		24.25 ± 8.86						
LDL-C (mg/dl)							*H* = 54.339^∗^	<0.001^∗^	0.025^∗^	<0.001^∗^	<0.001^∗^
Min.–Max.	19.0–129.0		45.0–360.0		112.0–345.0						
Median	97.50		113.50		197.0						
			
HbA1c (%)							*F* = 78.854^∗^	<0.001^∗^	<0.001^∗^	<0.001^∗^	0.013^∗^
Min.–Max.	4.0–6.20		6.80–10.10		7.0–12.20						
*X* ± SD	5.0 ± 0.71		8.05 ± 0.86		8.79 ± 1.45						
TNF-*α* (ng/l)							*H* = 64.775^∗^	<0.001^∗^	0.001^∗^	<0.001^∗^	<0.001^∗^
Min.–Max.	9.0–76.0		48.0–193.0		102.0–480.0						
Median	31.0		92.50		232.0						
ACR (mg/g)							*U* = 0.0^∗^	<0.001^∗^	—	—	—
Min.–Max.	—		6.0–29.0		31.0–535.0						
Median			18.50		61.50						

**Table 2:** Correlation between serum TNF-*α* levels and different parameters among DN.

	TNF-*α*	
	*r*_s_	*p*
BMI (kg/m^2^)	0.147	0.421
FBS (mg/dl)	0.362	0.042^∗^
2 h pp (mg/dl)	0.218	0.232
BUN (mg/dl)	0.004	0.982
Creatinine (mg/dl)	0.830	<0.001^∗^
TG (mg/dl)	0.317	0.077
Total cholesterol (mg/dl)	0.675	<0.001^∗^
HDL-C (mg/dl)	-0.288	0.110
LDL-C (mg/dl)	0.661	<0.001^∗^
HbA1c (%)	0.517	0.002^∗^
ACR (mg/g)	0.360	0.043^∗^

*r*_s_: Spearman coefficient. ^∗^Statistically significant at *p* ≤ 0.05.

**Table 3:** Comparison between the studied groups according to genotype distribution.

	Control group (*n* = 20)		T2DM group (*n* = 38)		DN group (*n* = 40)		*χ* ^2^	*p*
	No.	%	No.	%	No.	%		
Genotype								
TT	16	80.0	24	63.2	17	42.5	9.624^∗^	MC*p* = 0.037^∗^
TC	4	20.0	12	31.6	16	40.0		
CC	0	0.0	2	5.3	7	17.5		
Allele								
T	36	90.0	60	78.9	50	62.5	11.911^∗^	0.003^∗^
C	4	10.0	16	21.1	30	37.5		

*χ* ^2^: chi-squared test; MC: Monte Carlo; *p*: *p* value for comparing between the three groups. ^∗^Statistically significant at *p* ≤ 0.05.

**Table 4:** Odds ratio of genotypes and alleles.

	Controls vs. T2DM		Controls vs. DN		T2DM vs. DN		Controls vs. (T2DM+DN)	
	OR	95% CI	OR	95% CI	OR	95% CI	OR	95% CI
Genotype								
TT	1.000		1.000		1.000		1.000	
TC	2.000	0.55–7.31	3.765^∗^	1.03–13.69	2.039	0.78–5.36	2.732	0.83–9.04
CC	—	—	—	—	2.625	0.46–14.96	—	—
Allele								
T	1.000		1.000		1.000		1.000	
C	2.400	0.74–7.74	5.400^∗^	1.75–16.68	2.250^∗^	1.10–4.59	3.764	1.27–11.18

OR: odds ratio; CI: confidence interval.

**Table 5:** Multivariate logistic regression model for the risk factors of DN.

Parameters	Odds ratio	*p*	95% CI
BUN (mg/dl)	1.433	<0.001^∗^	1.213–1.693
Creatinine (mg/dl)	1544.6	<0.001^∗^	95.3–25021.3
TG (mg/dl)	1.062	<0.001^∗^	1.039–1.086
Total cholesterol (mg/dl)	1.029	<0.001^∗^	1.018–1.039
HDL-C (mg/dl)	0.864	<0.001^∗^	0.816–0.915
LDL-C (mg/dl)	1.028	<0.001^∗^	1.018–1.038
HbA1c (%)	1.654	<0.001^∗^	1.218–2.246
TNF-*α* (ng/l)	1.048	<0.001^∗^	1.026–1.071
C allele	2.446	0.035^∗^	1.065–5.616
Duration diabetes (years)	7.147	<0.001^∗^	3.056–16.715

CI: confidence interval.

## Data Availability

The data used to support the findings of this study are available from the corresponding author upon request.
